# HRGFish: A database of hypoxia responsive genes in fishes

**DOI:** 10.1038/srep42346

**Published:** 2017-02-13

**Authors:** Iliyas Rashid, Naresh Sahebrao Nagpure, Prachi Srivastava, Ravindra Kumar, Ajey Kumar Pathak, Mahender Singh, Basdeo Kushwaha

**Affiliations:** 1 Molecular Biology and Biotechnology Division, ICAR- National Bureau of Fish Genetic Resources, Lucknow- 226002, Uttar Pradesh, India; 2 AMITY Institute of Biotechnology, AMITY University Uttar Pradesh, Lucknow-226028, Uttar Pradesh, India; 3 Fish Genetics and Biotechnology Division, ICAR- Central Institute of Fisheries Education, Mumbai-400 061, Maharashtra, India

## Abstract

Several studies have highlighted the changes in the gene expression due to the hypoxia response in fishes, but the systematic organization of the information and the analytical platform for such genes are lacking. In the present study, an attempt was made to develop a database of hypoxia responsive genes in fishes (HRGFish), integrated with analytical tools, using LAMPP technology. Genes reported in hypoxia response for fishes were compiled through literature survey and the database presently covers 818 gene sequences and 35 gene types from 38 fishes. The upstream fragments (3,000 bp), covered in this database, enables to compute CG dinucleotides frequencies, motif finding of the hypoxia response element, identification of CpG island and mapping with the reference promoter of zebrafish. The database also includes functional annotation of genes and provides tools for analyzing sequences and designing primers for selected gene fragments. This may be the first database on the hypoxia response genes in fishes that provides a workbench to the scientific community involved in studying the evolution and ecological adaptation of the fish species in relation to hypoxia.

Hypoxia, a condition where the deficient amount of oxygen reaches the body tissues, is a common abiotic stress in fishes that generally occurs due to the increase in the water temperature, presence of organic pollutants as well as aquatic flora, which consume ambient oxygen, and lead to rapid reduction of the oxygen tension in the water^1^. Hypoxia has a direct effect on the physiological and cellular function of fishes^2^. The rate of diffusion of the atmospheric oxygen in water becomes worse when the water surface is covered by ice, thick vegetation or coral reefs^3^,^4^. The consistent degradation of oxygen level in the tissues affects the regulation and expression of oxygen dependent genes. Further, the studies on the hypoxia tolerance have shown the imperative role in the rate of the oxygen diffusion and its concentration in tissue in the evolution and ecological context of the aquatic organisms, including fish, that force them for environmental adaptation in variety of ways^5^. A kind of environmental adaptation is considered to be the variability in hemoglobin structure and function to transport more oxygen to various tissues of the body^6^,^7^,^8^. An adaptation of alternative metabolic pathways of anaerobic energy production was also reported in the *Carassius* species of the family Cyprinidae during extreme hypoxia in which fermentation of the glucose produces ethanol and carbon dioxide^9^,^10^,^11^. Zebrafish undergoes evolutionary adaptation during embryogenesis and able to grow with enhanced hypoxia tolerance in the subsequent developmental stages^12^. The storage of large amounts of glycogen helps the organism to adapt for the long term survival in the hypoxic condition^13^. In fishes, evolutionary adaptation is not uniform and physiology of several species significantly alters under hypoxic conditions^14^,^15^,^16^,^17^.

Several approaches were conceded for the identification of the hypoxia responsive genes in fishes and their altered expression levels. Reports also revealed that the nuclear transcriptional factor is responsible for inducing gene expression in the hypoxic condition at the site, which is subsequently required for transcriptional activation of human EPO gene enhancer^18^. The discovery of hypoxia inducible factor (HIF) and its involvement in gene regulation during the hypoxic condition has been observed in many genes encoding EPO, VEGF glucose transporter-1 (GLUT-1), heme oxygenase (HO), transferrin, inducible nitric oxide synthase (iNOS), and the glycolytic enzyme aldolase A, enolase 1, lactate dehydrogenase A, phosphofructokinase L and phosphoglycerate kinase I^19^. HIF is a highly conserved heterodimer composed of an alpha and a beta subunit of protein chain that responds to the changes of the oxygen tension in the cellular environment. Three hypoxia inducible factors, each with two isoforms, *viz*. HIF-1α, HIF-1β, HIF-2α, HIF-2β, HIF-3α and HIF-3β, have been reported^20^. Rainbow trout was the leading fish in which expression and function of HIF-1α were studied^21^. The HIF-1 binds with a cis-regulatory DNA sequence, called hypoxia response element (HRE), in several genes reported for enhancing mRNA formation during the hypoxia^22^. Functional HREs are being reported in several mammalian genes^23^, while few studies have proposed functional HREs in fishes^24^,^25^.

Many genes characterized in response to the hypoxia in fishes are available in the public domain. Prior to this study, hardly any effort was made to compile and organize such genes in the form of a centralized digital resource for the fishes. To meet this, a database for hypoxia responsive genes in fish (HRGFish) was developed to collect, organize and manage the genes and gene information. HRGFish is a platform for analyzing genes and gene sets for evolutionary and diagnostic studies. The different sets of reported genes for hypoxia response were identified by screening the published research articles, and information including Gene Ontology (GO) terms were taken from on-line published sources. Linux Apache MySQL PHP and Perl (LAMPP) technology was used to create the database, design and connect the data-driven web pages integrated with browse, analysis and view functionalities. *In silico* approaches were applied to identify the upstream sequences, promoter regions and different sets of orthologous promoters from the genes in the database. An analysis of upstream region can be useful in understanding the gene regulation, regulatory elements, hypoxia response element (HRE) orientation, tolerance and the susceptibility of the species against hypoxia. The different types of analytical tools were included in the web interface of the database for analyzing the gene sequences and designing primer of the selected region. It is expected that the HRGFish resource might fill the knowledge gap in understanding the cellular adaptation in response to the hypoxia and the role of the intra- as well as inter-specific genetic variation in hypoxia tolerance.

## Results

**Figure 1:**
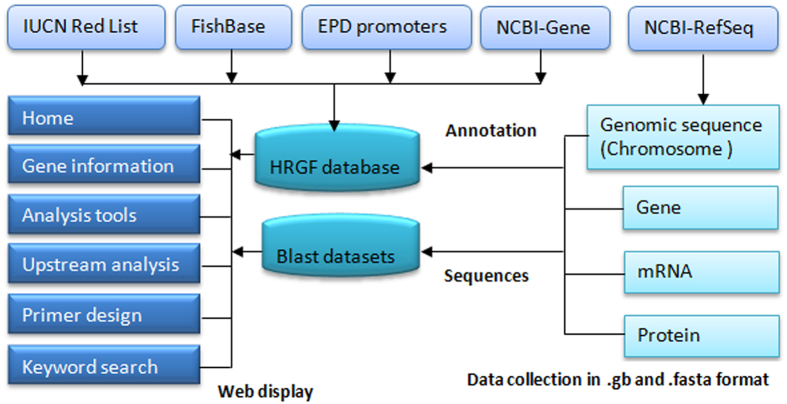
Architecture and data flow diagram of HRGFish.

HRGFish presently covers a total of 818 gene sequences for 35 types of genes belonging to 38 fish species and is now freely accessible at URL: http://mail.nbfgr.res.in/HRGFish. The web based workbench of HRGFish includes menu items for retrieving, browsing and analyzing gene information and sequences. Tools for upstream and downstream analysis, primer design, sequence similarity and keyword searches have been integrated that enhance the utility and visibility of the database (Fig. 1).

### Browsing gene information

**Figure 2:**
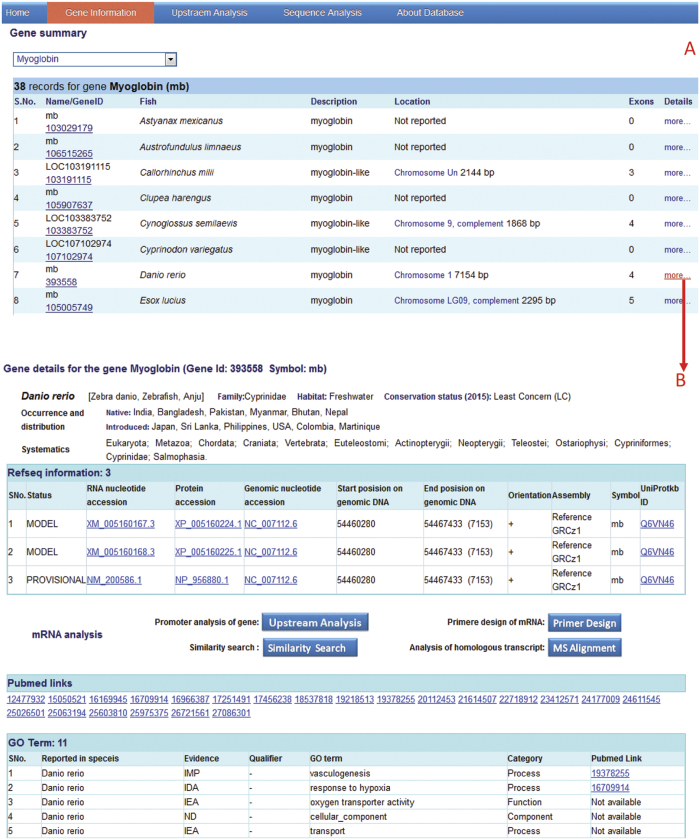
Web interface of HRGFish database: (A) display orthologous gene in fish species, and (B) details information about the gene and fish species.

The web interface of HRGFish contains ‘Gene Information’ menu item that provides the ability to view the list of genes and the detailed information about the gene of interest. The selection of a gene from the list of genes displays all the orthologous genes in a table, presenting the gene name, description, chromosomal information, genomic accession, gene location, number of exons reported in the gene and the species name (Fig. 2A). Each row in the table of orthologous genes contains a hyperlink ‘more’ in the last column. A click event on this link opens up the ‘genedetails’ page. The ‘genedetails’ page provides more information about the gene (chromosome, mRNA, protein, GO and PubMed) and species (family, common name, synonyms, habitat, conservation status, occurrence, distribution and taxonomy) in different sections. The RefSeq gene information section presents IDs for mRNA variants, protein, genomic DNA, uniprot and localized region of genes and mRNAs in genomic DNA. The Pubmed provides a table of publications related to gene and the gene ontology section defines the go term of a gene like location, process and function of the gene product. All IDs presented on this page have links to their respective databases for cross validation of information. This page includes tools for different activities like: i) designing primer that uses gene/mRNA as a query of the selected region of the gene, ii) analyzing orthologous promoters of the gene, similarity analysis of the gene sequence of interest with sequences of the database, and iii) multiple sequence alignment analysis to observe the conserved regions across the species. The primer designing facility for selected exonic regions has been included for experimental validation by PCR (Fig. 2B).

### Knowing about the database

The ‘About Database’ menu item in the web interface provides a graphical and textual description of tools included in the interfaces. This menu item also gives detailed information about the database and the tools to be used for browsing, retrieving and analyzing the information from the database.

### Keyword search

The keyword search accepts keywords like species name, gene name, gene symbol, accession number, transcript ID and PubMed ID as input for retrieving records from the database. Different views for the different keywords have been prepared to display the information.

### Upstream information

The ‘Upstream Analysis’ menu item included a drop down option ‘Pattern analysis’ in the web interface, which provides the ability to compute CG dinucleotide frequency, HRE motifs and observation of CpG islands in the upstream sequence of the selected gene. The sequence string and distribution of different motifs, like CG and HRE, are presented in graphical form, while CpG islands observation draws a graphical plot. The similarity search analysis of the upstream sequences with available reference promoters of the selected gene provides the common conserved patterns within the set of the orthologous promoters.

**Figure 3:**
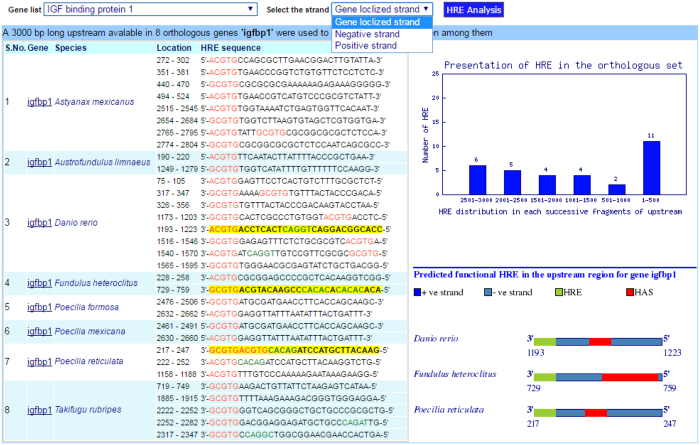
The HRE analysis page: Presenting all HRE in the orthologous set of IGFBP1 gene. Bar diagram presents the frequency of HRE in each 500 nt long consecutive fragments of upstream, while graphical plots display the functional HRE in gene localized on the strand.

The ‘Upstream Analysis’ menu item also contains another drop-down option ‘HRE analysis’ which provides a separate interface for HRE analysis of all the orthologous genes. This module mines all HREs from the set of the orthologous upstream of a selected gene and the selected orientation and presents: (i) a table of all HREs and yellow highlighted predicted functional HRE, (ii) a graph of HRE distribution for each 500 nt long consecutive fragment, and (iii) a graphical plot of predicted functional HREs. This module facilitates the mining of canonical HRE from the sequence, including forward and reverse orientation in the set of the upstream of a selected gene (Fig. 3).

### Primer design

The ‘Sequence Analysis’ menu item includes a submenu item ‘Primer design’ that uses a query interface. The query interface accepts input from the user and the result of query provides the details about the designed primers in a graphical format for both forward and reverse primer sets with a suitable length of flanking region. Additionally, the page presents information about the query specimen, selected subsequence and multiple primers with Tm values, GC content, start to end position and PCR product size. These designed primer sequences could be useful for analyzing and obtaining homologous promoters and exons of a gene across the species through PCR.

### Sequence alignment

The ‘Sequence Analysis’ menu item in the HRGFish facilitates many options for analyzing the query sequences against the HRGFish database. The pairwise sequence similarity searching can be done through ‘Similarity search’ listed under ‘Sequence Analysis’ menu item and from the ‘genedetails’ page also. There are two methods to perform the alignment. One method is that the user selects any internal gene, mRNA and protein sequence as an internal query sequence to perform alignment with ‘HRGFish’ database. Another method is pairwise alignment that can be performed using the query sequence in FASTA format along with input parameters. Four input types are required for performing the alignment: (1) the selection of a target dataset as listed in Table 1, (2) the selection of a dataset-compatible Blast algorithm, (3) the selection of a sequence (DNA or protein) and (4) an input sequence in FASTA format. User input parameters such as maximum alignment number, identity percent cut off and word size can also be provided using Blastn, whereas Blastp and Blastx program use only the default parameters. The acceptance of these inputs through the submit button performs an alignment with the target sequences. If parameters are not selected correctly, the program terminates by giving a warning message. Thus, HRGFish platform provides the ability to the user for analyzing gene sequences, segments of a sequence, translated products and annotate genes in the newly reported fish genome.

Apart from the different blast program for homologous sequence alignments, HRGFish also includes other useful programs under alignment analysis for specific search. The implemented program ‘Spidey’ provides the ability to the user to align mRNA sequence with the genomic sequence and supports to identify the transcribed regions and annotation information of a gene in the genomic sequences. The ORF finder program computes open reading frames for the user provided query mRNA as input. This program helps in the gene annotation process. In this way, these programs provide the ability to the user for gene identification and annotation obtained from exon-intron orientation in related species. The multiple sequence alignment program ‘ClustalW’ was implemented in HRGFish for analysis of conserved patterns among homologous mRNA sequences and obtaining orthologous clusters within a set of selected sequences.

## Discussion

A number of hypoxia response genes have been characterized in fishes, but the attempts were lacking to compile and organize the information on a platform for collectively viewing and analyzing these data^2^. Hence, Hypoxia Responsive Genes in Fish (HRGFish) was developed to organize and manage the genes and their related information. The gene, upstream region and mRNA sequences were collected directly from the corresponding genomic sequences and reverse complement parsing methods were applied to the genes reported on the negative strand. Some analytical tools were implemented to analyze the gene, mRNA and the upstream sequences.

Orthology based promoter prediction and recognition of regulatory site in the upstream is an effective approach for promoter annotation^26^. The same approach was also applied here to display the orthologous set of promoter sequences across related species using reference promoter of the same gene of zebrafish. The localization of the binding sites for transcription factors provides support for promoter recognition in eukaryotes^27^. CpG islands are short interspersed DNA fragments enriched with dinucleotide motifs CG and found in or near the promoters of a gene and form transcription factor binding sites (TFBSs)^28^. In the present study, the observation of conserved motifs and analysis of CpG islands in the upstream region confirms the presence of the regulatory elements, like enhancer and promoter of a gene. In this way, the CpG island identification in the upstream region provides a support to identify the presence of TFBSs and annotation of the promoter regions.

The gene involved in the hypoxia response contains HRE elements for HIF-1 binding, which are upregulated during the hypoxia. HREs having functionally essential hypoxia binding site consists of the consensus sequence described in 5′ upstream enhancer and promoter regions and 3′ downstream of the 3′ UTR^29^. The functional HRE have been identified in several human genes that are associated with a HIF-1 binding site (5′-(A|G)CGTG-3′) and its HIF-1 ancillary sequence (HAS), 5′-CA(G|C)(A|G)(T|G|C)-3′, located at downstream^23^. But, reports on the functional HRE in fish was not available broadly. A novel HRE, 5′-GATGTG-3′, was reported inside the second introns in the lactate dehydrogenase-B gene in killifish^25^. The functional HRE in zebrafish have been reported for the IGFBP-1 genes along with HRE consensus sequence (5′-RCGTG-3′) and its HAS sequence (5′-CAGGT-3′) in the downstream spacing 15 nt^30^. The upstream sequences were taken for HRE analysis, and the approaches of Kimura’s and Kajimura’s were applied here for analyzing the upstream region of hypoxia response genes in fishes. An HRE sequence (5′-RCGTG-3′) and consensus HAS [5′-CA(G|C)(A|G)(T|G|C)-3′], located in a space of 7–15 nt downstream, were considered to determine the putative functional HRE from canonical sequences of upstream.

This platform has also a primer design module which may be used to design primers of the locating region of functional HRE on the upstream sequence for experimental validation.

The presence of HRE with an adjacent motif pattern of HAS in regulatory elements can be used as a marker to identify the gene induced by HIF-1 in hypoxic condition. The homology based annotation and findings of CpG islands and HRE pattern in the parsed upstream sequences support the existence of the promoter and enhancer regions.

The cross species PCR (cs-PCR) amplification was applied frequently to obtain common regions of a gene sequences among related species. Iyengar^31^ applied cs-PCR approach to amplify the region of interest into four species of non-human primates. The proposed work will help to design primers for amplification of the conserved exonic region of a gene to validate homologous gene in different fish species. Further, the reported genes for hypoxia response in zebrafish and other model fishes will help to identify the homologous genes in hypoxia susceptible and tolerant species using the cs-PCR, which may provide a greater insight into the stress management in the fishes.

## Materials and Methods

### Data collection and parsing

The peer-reviewed research articles were extracted from the NCBI’s PubMed using keywords, like hypoxia, anoxia etc., in fishes. The shortlisted articles from the search result were used for identifying the hypoxia responsive genes in fishes. Table 2 presents a list of genes identified from the shortlisted articles for hypoxia response in fishes. The information about the identified genes (Table 2) were collected from the Gene database^32^. For parsing and managing data in the HRGFish database, a Perl parsing program (GeneDB_Mapper.pl) was designed and implemented for parsing GeneID, gene ontology terms, PubMed ID and Accession ID of mRNA, protein and gene containing chromosome (genomic sequence) from the Gene database according to the database schema. The accession numbers for mRNA, protein and gene containing genomic sequences for the reported genes in the Gene database were collected and supplied in the Batch Entrez of NCBI^33^ and the data files in the FASTA format were downloaded. After that, another Perl script ‘GeneSeqParse.pl’ was designed and implemented for parsing gene and mRNA sequences by using their position of localization on the downloaded genomic sequences. An additional attempt was made to parse genomic mRNA sequences, because the downloaded mRNA from the curated database of NCBI had only coding regions (exonic) existing in the form of different variants in many genes which provided limited scope for analysis. On the other hand, the mRNA sequences with reported exons and introns were found much applicable for analysis and designing primers. Both types of mRNA sequences were taken into account for the study in different kind of analysis.

Both Perl scripts support the inclusion of a new hypoxia responsive gene and gene information for updating HRGFish database. The gene information of the species was enriched with general information (taxonomy, habitat, occurrences, distribution) collected from FishBase^34^ and conservation status collected from IUCN^35^. The modus operandi applied in the Fish Karyome^36^ was used for integration of genomic data with other fisheries information. Figure 1 presents the complete architecture of the HRGFish database.

### Identification of upstream elements

The upstream regions of different genes were collected. An orthologous cluster of upstream regions was formed and analyzed to deduce the conserved pattern of promoters. To carry out this work, genomic sequences of the reported gene for parsing the upstream sequence and a reference fish species, like zebrafish, in which promoter sequences are available, were essentially required. The EPD^37^ database was used to obtain promoter sequences of the zebrafish for the reported hypoxia responsive genes covered in HRGFish. Moreover, 3000 nt long upstream fragments located at −2900 to 100 position of the gene start site on genomic sequences were parsed. The upstream sequence of the selected genes was used to compute CpG (5′-C_phosphate_G-3′) island using CpG Ploter, HRE and HAS motif pattern. Further, alignment analysis of upstream sequences with reference promoters of zebrafish was done to deduce conserved patterns. These conserved patterns formed numerous clusters in the form of an orthologus set of promoter sequences for several genes.

### Database development

MySQL, a relational database management system, was used to design and implement the database under the Linux operating platform on Intel Xeon based high performance computer server machine. Tables were designed and relationships between the tables were created using unique, primary and foreign keys. Tables ‘fishinfo’ and ‘taxonomy’ cover general information on the fish species and their systematics respectively. Tables ‘genesummary’, ‘gene2refseq’, ‘gene2go’ and ‘gene2pubmed’ contains data collected from Gene database. Tables ‘rna’ and ‘cds’ cover annotation information on genes. The FASTA format files of gene, mRNA and protein sequences were used by the program ‘formatdb’ of the Blast suite for building the local Blast-compatible target datasets to perform alignment between the query and the target sequences (Table 1). The Blast-compatible datasets were synchronized with the main database and getting updated automatically when the main database is updated.

### Design and development of web interfaces

For web-based information delivery and analysis, user interactive web pages were designed and implemented using PHP (pre hypertext processor), Perl, DBI (database interface), CGI (common gateway interface), GD (graph display), Ajax, JavaScripts, CSS (cascading style sheets) and HTML technologies.

### Implementation of tools for analysis

Different tools were implemented in the web interface to work with the HRGFish database. The web interface integrated with these tools provides the workbench for searching, browsing and analyzing sequences from the database (Fig. 1). The similarity search tools, like BLAST, Spidey^38^ and GetORF^39^, were implemented for analyzing the homologous sequences, CDS, mRNA and Open Reading frames in the gene sequences. ClustalW2^40^ program was implemented for aligning a set of selected homologous sequences to analyze the conserved pattern among them. The primer3^41^,^42^ program was implemented for designing primers using methodologies already described in FishMicrosat^43^ and FMiR^44^. The CpGplot^39^ was implemented to analyze the CpG island in the upstream region of the gene.

## Conclusion

The primary goal of the HRGFish database is to serve as a repository of genes involved in hypoxia response in fishes and to provide a workbench for browsing information and analyzing the gene sequences. In addition, this platform provides the facility for primer designing for amplifying a selected region of a gene, which is applicable to identify the homologous sequence in other closely related fish species based on cross-species PCR amplification. HRGFish further highlights the annotation of the upstream sequences of genes for observation of HREs, TFBSs and other conserved elements of the promoter sequences which may play an important role in gene expression during hypoxia exposure. The findings of the HRE in the upstream regions of a gene confirm its expression that might be accelerated by a HIF transcription factor during hypoxia. Apart from that, the database contains taxonomy and general information of each fish species for categorization of the species and group-wise orthologous analysis of the promoter clusters. In future, the size of the database will grow by adding the records of new genes associated with hypoxia response. In this way, HRGFish is a prominent resource covering useful information and analysis tools for the cutting edge research areas related to the study of the hypoxia response and its applications in fishes, such as genetic relatedness among the species and genetic improvement programs of commercially important aquaculture species.

## Additional Information

**How to cite this article**: Rashid, I. *et al*. HRGFish: A database of hypoxia responsive genes in fishes. *Sci. Rep.* **7**, 42346; doi: 10.1038/srep42346 (2017).

**Publisher's note:** Springer Nature remains neutral with regard to jurisdictional claims in published maps and institutional affiliations.

## Figures and Tables

**Table 1:** Different Blast-compatible datasets.

Dataset name	Description	No. of sequences	Compatibility
upstrmdb	Upstream sequences of genes	799	Blastn
genedb	Genes sequences	799	Blastn
protdb	Protein with isoformes sequences	1413	Blastx, Blastp
mrnadb	Exonic sequences of mRNA and its varients	1424	Blastn
genomicmrnadb	Genomic mRNA sequences along with varients	1414	Blastn

**Table 2:** Hypoxia response genes reported in fishes.

S. No.	Gene name	Gene symbol	Pubmed IDs	Total genes in HRGFish
1	Aldolase A	aldoa	8955077, 21266200	13
2	Calcium/Calmodulin-dependent protein kinase	camk2g	15821280, 25840431	9
3	Ceruloplasmin	cp	15741220	39
4	Connective tissue growth factor	ctgf	25455470	30
5	CREB binding protein	cbp	8917528, 17925579	23
6	CREB regulated transcription coactivator 3	crtc3	25840431	22
7	E1A binding protein p300	ep300	8917528	14
8	Endothelial PAS domain protein 1	epas1	25205386	31
9	Enolase 1	eno1	8955077, 21266200, 26762295	24
10	Erythropoietin	epo	21798198, 17579187	51
11	Ferritin	fth1	11172064	42
12	Fibrillarin	fbl	17828398	27
13	Glucose transporter 1	slc2a1	10401038, 18941827, 12846834	4
14	Glucose transporter 2	slc2a2	19162213	22
15	Glucose transporter 3	slc2a3a	16081168	1
16	Glucose transporter 4	slc2a4	16081168, 8941827	30
17	Heme oxygenase-1	hmox1	24780551	21
18	Heme oxygenase-2	hmox2	24780551	71
19	Hemopexin	hpx	11172064	20
20	Hypoxia induced factor 1 alpha	hif1a	11278461	35
21	IGF binding protein 1	igfbp1	16428465, 18769480, 21730259 21501614	9
22	IGF binding protein 2	igfbp2	22639285	25
23	IGF binding protein 3	igfbp3	18769480	9
24	Lactate dehydrogenase B	ldhb	19439190, 11441966	24
25	Myoglobin	mb	25026501, 25595439	38
26	Noelin	noelin	17828398	8
27	Notch 2	notch2	17828398	5
28	P300-interacting transactivator	cited1	8917528, 20161383, 20547241	21
29	Peroxisome proliferator activated receptor alpha	ppara	25869933, 25205386, 25543049	27
30	Titin	ttn	11788825	12
31	Transferrin	tf	17646932, 11172064, 9242677	12
32	Vascular endothelial growth factor A	vegfa	22300081, 15177948	30
33	Vascular endothelial growth factor B	vegfb	22300081, 15177948	27
34	Vascular endothelial growth factor C	vegfc	22300081, 15177948	22
35	VEGF receptor	flt1	20335444	20
			Total gene information	818
